# Software-based planning of ultrasound and CT-guided percutaneous radiofrequency ablation in hepatic tumors

**DOI:** 10.1007/s11548-021-02394-1

**Published:** 2021-05-11

**Authors:** M. J. van Amerongen, P. Mariappan, P. Voglreiter, R. Flanagan, S. F. M. Jenniskens, M. Pollari, M. Kolesnik, M. Moche, J. J. Fütterer

**Affiliations:** 1 Department of Radiology and Nuclear Medicine, Radboudumc, Nijmegen, The Netherlands; 2 NUMA Engineering Services Ltd., Louth, Ireland; 3 Robotics and Mechatronics (RaM), University of Twente, Enschede, The Netherlands; 4 Institute of Computer Graphics and Vision, Graz University of Technology, Graz, Austria; 5 Department of Computer Science, Aalto University School of Science and Technology, Espoo, Finland; 6 Fraunhofer Institute for Applied Information Technology FIT, Sankt Augustin, Germany; 7 Department of Interventional Radiology, Helios Park-Klinikum Leipzig, Leipzig, Germany; 8 Department of Mathematics and Statistics, IIT Tirupati, Tirupati, India

**Keywords:** Liver, RFA, Simulation software

## Abstract

**Objectives:**

Radiofrequency ablation (RFA) can be associated with local recurrences in the treatment of liver tumors. Data obtained at our center for an earlier multinational multicenter trial regarding an in-house developed simulation software were re-evaluated in order to analyze whether the software was able to predict local recurrences.

**Methods:**

Twenty-seven RFA ablations for either primary or secondary hepatic tumors were included. Colorectal liver metastases were shown in 14 patients and hepatocellular carcinoma in 13 patients. Overlap of the simulated volume and the tumor volume was automatically generated and defined as positive predictive value (PPV) and additionally visually assessed. Local recurrence during follow-up was defined as gold standard. Sensitivity and specificity were calculated using the visual assessment and gold standard.

**Results:**

Mean tumor size was 18 mm (95% CI 15–21 mm). Local recurrence occurred in 5 patients. The PPV of the simulation showed a mean of 0.89 (0.84–0.93 95% CI). After visual assessment, 9 incomplete ablations were observed, of which 4 true positives and 5 false positives for the detection of an incomplete ablation. The sensitivity and specificity were, respectively, 80% and 77% with a correct prediction in 78% of cases. No significant correlation was found between size of the tumor and PPV (Pearson Correlation 0.10; *p* = 0.62) or between PPV and recurrence rates (Pearson Correlation 0.28; *p* = 0.16).

**Conclusions:**

The simulation software shows promise in estimating the completeness of liver RFA treatment and predicting local recurrence rates, but could not be performed real-time. Future improvements in the field of registration could improve results and provide a possibility for real-time implementation.

## Introduction

Currently, radiofrequency ablation (RFA) is frequently used as a curative ablative therapy for the treatment of hepatic malignant tumors [1–6]. Although RFA shows promising results regarding the local control HCC or colorectal liver metastases with low morbidity and mortality, it can be associated with a higher local recurrence rate and subsequent lower disease-free survival compared to surgery [7; 8]. Besides the experience of the physician [9], the significant mismatch between the expected ablation zone and the observed ablation zone is an important factor contributing to insufficient ablations [1]. Multiple causes exist for this mismatch. On one hand, due to heat-sink, location of hepatic tumors near major blood vessels can result in undertreatment [10]. On the other hand, overtreatment can occur in cirrhotic liver tissue due to the insulating properties of fatty tissue surrounding the tumor, causing the ‘oven-effect’ [11]. Real-time monitoring during an RFA treatment could help to drive treatment and is being researched [12], however, this is currently not available in the clinic.

A European research group, including our hospital, created the planning software “the RFA Guardian”, details surrounding the creation of this software are described elsewhere [13]. The RFA Guardian software performs registration of CT images between different time points using defined landmarks for dynamic registration. On the initial preprocedural CT, the liver and its vessels are automatically defined and meshed after which the tumor was manually segmented in three dimensions. In order to achieve insight into biological parameters of both the tumor and normal liver tissue for the simulation of the ablation, CT perfusion values are implemented in the software. Using the CT-scan obtained during the procedure, the RFA needle can be virtually placed in the registered liver and the ablation can be simulated, visualizing possible over/undertreatment [13]. Using this planning software, the most important factors contributing to local recurrence rate, besides tumor size, e.g., experience of physician and mismatch expected zone and observed zone, are tackled.

The RFA Guardian was recently tested in a multicenter clinical study for the treatment of colorectal liver metastases (CRLM) and hepatocellular carcinoma (HCC) [14], describing patient selection, workflow and periprocedural imaging of the included patients. This earlier study compared the size and shape of the simulated ablation volume acquired by the software with the RFA ablation zone on the CT-scan one month after the procedure. Acceptable speed and accuracy of the simulation software were demonstrated in predicting the size and shape of the RFA ablation zones in the liver [14]. However, during the ablation procedure, the most important question remains whether the ablation was sufficient and covers the entirety of the tumor. For this purpose, the patient data provided by our hospital in the multicenter clinical trial [14] were re-evaluated whether the RFA Guardian software was retrospectively able to estimate the completeness of hepatic radiofrequency ablations and is therefore able to predict local recurrence rates.

## Materials and methods

### Patients

**Fig. 1:**
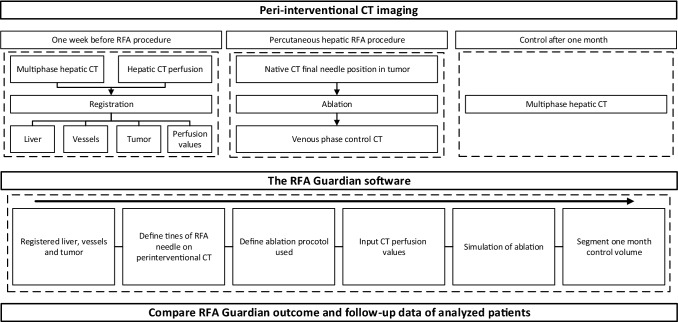
Workflow

Patient data provided by our hospital to a multicenter clinical trial analyzing the RFA Guardian [14] were re-evaluated. Trial approval for the initial study was granted by our Institutional Review Board (CMO region Arnhem—Nijmegen, Radboudumc, Nijmegen, The Netherlands). At our institution, AASLD guidelines are used in the tumor boards in order to determine the optimal treatment for presented patients. All patients were discussed prior to treatment in these multidisciplinary tumor boards. The initial study design is previously described and can be found elsewhere [14, 15]. In short: patients were included if they (1) were older than 18 years, (2) had a diagnosis of CRLM or HCC according to AASLD guidelines, (3) showed a maximum of 3 lesions with a maximum diameter of 3 cm each, (4) sufficient coagulation parameters, adherent to ESIR guidelines. Patients were excluded if they (1) rejected the trial, (2) had a known anaphylactic reaction against the iodine contrast agent used for diagnostic CT-scans, (3) showed malfunction of the kidney (Glomerular Filtration Rate < 60 ml/min/1.73m^2^), (4) history of prior splenectomy, (5) were pregnant or nursing or (6) participated in other interventional trials. Workflow with periprocedural images and planning steps of the RFA Guardian are depicted in Fig. 1.

In total, 27 patients were included from April 2014 until July 2017 for the evaluation of “the RFA-guardian” simulation software in a European project [13]. Of these patients, 18 were male and 9 patients were female with a mean age of 68 years (95% CI 64–72). CRLM was shown in 14 patients and HCC in 13 patients. A total of 32 lesions required ablation, due to technical difficulties, e.g., failure in processing CT perfusion data or software failure, only 27 tumors could be simulated and were evaluated. The mean size of the tumor was 18 mm (95% CI 15–21 mm). Patient demographics are summarized in Table 1.

**Table 1:** RFA protocol

Protocol type	Needle deployment (cm)	Target temperature	Power (W)	Duration
3 cm ablation size	2	105 °C	150	Until target temperature achieved
	3	105 °C	150	5 min at target temperature
4 cm ablation size	2	105 °C	150	Until target temperature achieved
	3	105 °C	150	Until target temperature achieved
	4	105 °C	150	7 min at target temperature
5 cm ablation size	2	105 °C	150	Until target temperature achieved
	3	105 °C	150	Until target temperature achieved
	4	105 °C	150	7 min at target temperature
	5	105 °C	150	7 min at target temperature

Ablation was always started at a 2 cm needle deployment. When the duration of the ablation of specified deployment was achieved, the needle was further deployed. If the protocol was deemed complete, the RF generator automatically performed its cool-down procedure before track ablation could be performed

### Preprocedural imaging and software planning

According to the study design of the multicenter clinical trial [14], patients received a diagnostic multiphase hepatic CT with added dynamic CT measurements after contrast administration within 7 days prior to the treatment for planning purposes in the RFA Guardian software. Because the liver moves and deforms during breathing, all CT-scans were performed during expiratory breath-hold in order to achieve the most reproducible image of the liver in multiple time-points. The RFA Guardian software generated a complete 3D model of the liver and segmented the arterial and venous vessels in the liver. During this process, the hepatic tumor was segmented by a radiologist. The steps necessary for treatment planning in the RFA Guardian are described elsewhere [13].

### Radiofrequency ablation

Percutaneous RFA was performed by either one of two interventional radiologists, both with more than 10 years of experience, and was conducted on a CT-table with the use of an umbrella-shaped RF probe (Starburst™ SDE, AngioDynamics, USA) and an RF generator (RITA^®^ Model 1500X RF, AngioDynamics, USA). During the procedure, an ultrasound device was available. The procedure was performed under general anesthesia. If the lesion was easily visualized with ultrasound, preference was given to this modality; otherwise, unenhanced CT was used for placement guidance. When the needle position in the liver was deemed optimal for ablation, an unenhanced CT was performed to identify the needle location for simulation process in the RFA Guardian [13]. The ablation commenced after the unenhanced CT-scan of the needle position. Ablation adhered to the liver tumor protocol as provided by the manufacturer, see Table 2. If multiple ablations for a single tumor were deemed necessary, the previous steps were repeated. After the procedure, the needle was removed under track ablation and a final contrast-enhanced CT was performed to visualize the performed ablation.

**Table 2:** Patient demographics

	Patients undergoing liver ablation (*n* = 27)	
Age (mean [95% CI])		68 years (64–72)
Gender (M: F)		18 male: 9 female
ASA classification	ASA 1	2 patients
	ASA 2	12 patients
	ASA 3	9 patients
	ASA 4	4 patients
Type of liver tumor (CRLM: HCC)		14 CRLM: 13 HCC
Number of lesions		32 lesions
Number of simulations		27 simulations
Mean tumor size (96% CI)		18 mm (95% CI 15–21 mm)

### Post-procedural

After the ablation, patients stayed in the hospital overnight due to the given anesthesia and to check for possible post-procedural hemorrhage. The ablated region was given time to allow for tissue shrinkage after the treatment. For this reason, all patients received a multiphase hepatic CT-scan for HCC and mono-phase CT (portal phase) for CRLM during expiratory breath-hold after a month after the ablation for the evaluation of the ablation. On this control CT-scan, an abdominal radiologist visually evaluated the size and position of the ablation and compared this with the preprocedural CT scan. If the tumor was deemed completely covered by the ablation zone, the procedure was deemed complete. Further follow-up was performed by the referring physician. Local recurrence was defined as recurrent tumor at the ablation site during follow-up.

### Simulation and post-processing

**Fig. 2:**
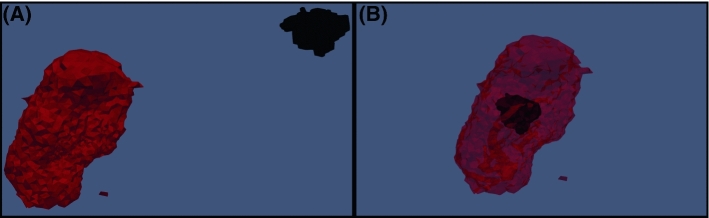
Overlap of the tumor by the simulated lesion simulated volume (red) and tumor (black) before and after correction of registration error.** a** Overlap before correction,** b** Overlap after proper correction of the registration error

The RFA Guardian performs calculations using state-of-the-art graphics processing unit (GPU) [16]. The parameters for simulation are estimated by an algorithm involving a proportional-integral-derivative (PID) controller. The development and performance of this simulation algorithm are described previously [16]. The liver containing the simulated ablation volume generated by the RFA Guardian software on the periprocedural unenhanced CT-scan was retrospectively co-registered to the liver containing the initial tumor on the preprocedural CT images. The overlap between the tumor volume and simulated ablation zone was calculated using positive predictive value (PPV) and ranged between 0 (tumor is not overlapped with simulated volume) and 1 (tumor is completely covered with the simulated volume). Also, an additional visual assessment was used for this overlap since simulated vessels can cause gaps where there is sufficient ablation, lowering the calculated PPV. These values were computed after correction for co-registration errors by picking eight landmarks of the hepatic surface [16]. These co-registration errors are probably due to liver deformity during multiple image acquisitions at different time-points, specifically, the differences in expiratory breath-hold during preprocedural images and end-expiratory apnea during general anesthesia in the preprocedural CT images and are visualized in Fig. 2.

### Statistical analysis

Patients demographics, ablation and simulation details were analyzed using means and ratio determination. Frequency of local recurrence after ablation was summarized. Length of follow-up was calculated and in case of a recurrence, the possible treatment (palliative/curative) was noted. Correlation between the size of the tumor and PPV was calculated using Pearson’s r. Correlation between PPV, recurrence rate and size of the tumor was calculated with the use of one-way ANOVA and Pearson’s r. Disease-free survival (DFS) and overall survival (OS) were calculated using the Kaplan–Meier estimator. SPSS software (IBM SPSS Statistics, version 20, SPSS, Chicago, Ill) was used for data processing and analysis. Significance difference was deemed if the *p* value was less than 0.05.

## Results

### Post procedural outcome

Most patients could be discharged the day after the ablation. A total of 3 complications (11%) were observed, i.e., a transient ischemic attack (Clavien-Dindo Grade 2), pneumothorax (Clavien-Dindo Grade 2) and an abundance of pleural fluid in a cardiologic patient (Clavien-Dindo Grade 2). None of these complications resulted in permanent harm for the patient. During a mean follow-up of 18 months (95% CI 14–22 months), 5 recurrences at the ablation site were shown (19%), meaning a sensitivity and specificity of the one-month control CT scan of, respectively, 0% and 100%. The size of the tumor (maximum of 30 mm) did not show a significant correlation with local recurrence rates (Pearson Correlation − 0,05; *p* = 0.79).

### Simulation outcome

Mean simulation time of the lesions was 2.9 min (2.0–3.8 95% CI). The positive predictive value (PPV) regarding tumor and simulated lesion showed a mean of 0.89 (0.84–0.93 95% CI). After the visual assessment, only 9 tumors were not covered by the simulated lesions. Of the 5 patients with local recurrence after ablation, 4 cases were predicted using the software. However, five additional ablations were deemed incomplete, which did not show a local recurrence during follow-up, meaning, the software was able to predict the completeness of the ablation in 78% of cases (sensitivity 80% and specificity 77%). There was no significant correlation between the size of the tumor and the PPV (Pearson Correlation 0.10; *p* = 0.62) or between PPV and recurrence rates (Pearson Correlation 0.28; *p* = 0.16).

## Discussion

This study demonstrated that the registration and simulation software shows a high overlap between the tumor and simulated ablation (PPV 0.89 (0.84–0.93 95% CI) and was able to predict the completeness of the ablation in 21 cases of the 27 ablations (sensitivity 80% and specificity 77%).

In the prior multicenter clinical trial [14], an average absolute error (AAE) of 3.4 ± 1.7 mm was demonstrated between the simulated ablation volume and the “real” ablation volume at the one-month control CT-scan, proving to be acceptable for clinical practice. However, no prior research was available concerning whether the RFA Guardian was able to predict the local recurrence rate after hepatic RFA ablation. For this purpose, the included 27 ablations from our hospital were re-evaluated. The overlap between the simulated volume and the tumor showed a high PPV-value where 1 was complete coverage and 0 was no overage (PPV 0.89 (0.84–0.93 95% CI). However, because the software simulates major blood vessels in the liver for the purpose of heat-sink, gaps occur in the simulated volume, decreasing the automatically generated PPV-value. Therefore, an additional visual assessment was used in order to deem whether the simulation overlapped the tumor in the software and compared to the occurrence of a local recurrence during follow-up. All ablations were deemed complete at the one month control CT scan; however, in 5 patients, a local recurrence occurred during follow-up. The simulation predicted an incomplete ablation in 4 of these 5 insufficient ablations, however, with five incorrectly predicted insufficient ablations. Therefore, the simulation made a correct prediction in 21 of 27 ablations (78%) with a sensitivity of 80% and a specificity of 77. For this reason, the use of RFA Guardian for hepatic ablations has the potential to further improve oncological outcomes in patients, when in real-time results of the ablations can be visually assessed.

Registration errors play an important role within the fields of diagnostic imaging [17, 18]. The differences in the shape of the liver were deemed relevant in all perioperative images, although all CT-images were achieved during breath-hold (expiratory and apnea during general anesthesia). The liver deforms and alternates in the sagittal plane during breathing, making the organ different in each subsequent CT-scan. Landmarks can be used in order to improve the registration. However, this method makes the tissue between the landmarks fluid in order to fit the organ in the same contour over multiple timelines, deforming/displacing the tumor, RFA needle and simulated volume. In order to decrease these co-registration errors, additional re-registration was necessary. Though re-registration has its own limits, it does not affect the shape or size of the simulation, as these are calculated from a single CT time point and are not susceptible to registration.

This study has some limitations, and a major limitation was the number of patients included. Another limitation is the re-registration itself. Results before the re-registration reflect the reality of the clinical practice more accurately and show the difficulty of the registration of CT-scans between multiple time-points, especially because different CT-scanners can be used and placement of the patient on the CT table is always different. Re-registration with underlying transformation makes it possible to get around displacement difficulties and places the hepatic tumor and simulation in the same coordinate system, increasing statistics but losing the insight into the problems of normal registration. However, deformation of the liver during breathing still occurs and produces errors, causing the PPV value to decrease. Lastly, this retrospective study defined a successful ablation when the ablated volume showed a complete coverage of the tumor, omitting a safety margin. In future prospective studies, a safety margin of at least 5 mm needs to be included in the software in order to further decrease local recurrences.

Future work should be focused on investigating and improving the registration of CT-scans between multiple time-points. This will relieve the need for additional retrospective re-registration and will enable real-time usage of the simulation software during the RFA procedure. Separately, robotic assistance for needle placement during hepatic ablations has been developed and different systems are currently being researched [19–21]. For the use of these systems, CT-scans during the procedure are performed by temporary tube disconnection after which a needle trajectory and entry point are defined. A robotic arm is automatically positioned over the patient and the needle can manually or automatically be placed in the hepatic tumor according to the defined trajectory [19, 20]. High accuracy of these systems has been shown with a high rate of technical success of the procedure [19, 22]. Additionally, in order to decrease the impact of free hand placement of the RFA probe, stereotactic radiofrequency ablation (SRFA) with multiple probes has been developed [23]. Using 3D stereotactic software, optical navigation systems and vacuum fixation systems, this technique safely and reliably achieves favorable therapy outcome, although, a direct comparison between SRFA and surgery has yet to be performed [23–25]. Also, a possible higher accuracy can be achieved if the liver moves minimally during the entire ablation by avoiding possible deformation of the liver during the multiple peroperative time-points resulting from transiently pausing the mechanical ventilation. High-frequency ventilation (HFV) encompasses multiple ventilator modes with high respiratory rates and low tidal volumes, lowering the movement of thoracic and abdominal organs during general anesthesia [26]. In patients undergoing single-dose irradiation of liver tumors, liver motion was limited to < 3 mm in all directions with this ventilation technique [27]. Possibly, in the future, the combination of simulation software, robotic needle placement different ventilation techniques and use of stereotaxy could improve hepatic ablations by preventing under- or overtreatment with lower recurrence rates.

In conclusion, the simulation software shows promise in estimating the completeness of the liver RFA treatment and predicting local recurrence rates. In the future improved and faster registration between multiple CT-scans and the intervention will possibly further improve upon this, such as performing the task real-time during the ablation without the need of additional re-registration.

## Data Availability

DRadboudumc does not have a central access committee yet. Therefore, data request can be send to the corresponding author (Martin.vanamerongen@radbodumc.nl). Upon receiving a request, the corresponding author will evaluate the request with the Radboudumc dpt. of Medical Imaging's Research Office (trialbureau.radng@radboudumc.nl). If deemed necessary, the Institutional Ethical Review Board (CMO region Arnhem-Nijmegen) will be consulted.

## Abbreviations

RFA: Radiofrequency ablation
PPV: Positive predictive value
CI: Confidence interval
CRLM: Colorectal liver metastases
HCC: Hepatocellular carcinoma
CT: Computed tomography
GPU: Graphics processing unit
PID: Proportional-integral-derivative
AAE: Average absolute error
HFV: High-frequency ventilation
